# Identification of *Chlamydia trachomatis* CT621, a protein delivered through the type III secretion system to the host cell cytoplasm and nucleus

**DOI:** 10.1111/j.1574-695X.2009.00581.x

**Published:** 2009-08-14

**Authors:** Anne-Sofie Hobolt-Pedersen, Gunna Christiansen, Evy Timmerman, Kris Gevaert, Svend Birkelund

**Affiliations:** 1 Department of Medical Microbiology and Immunology, Aarhus UniversityAarhus C, Denmark; 2 Department of Medical Protein ResearchVIB, Ghent, Belgium; 3 Department of Biochemistry, Ghent UniversityGhent, Belgium

**Keywords:** *Chlamydia trachomatis*, secretion, type III secretion, subcellular proteome extraction, CT621 (CTL0885), two-dimensional gel electrophoresis

## Abstract

Chlamydiae are obligate intracellular bacteria, developing inside host cells within chlamydial inclusions. From these inclusions, the chlamydiae secrete proteins into the host cell cytoplasm. A pathway through which secreted proteins can be delivered is the type III secretion system (T3SS). The T3SS is common to several gram-negative bacteria and the secreted proteins serve a variety of functions often related to the modulation of host signalling. To identify new potentially secreted proteins, the cytoplasm was extracted from *Chlamydia trachomatis* L2-infected HeLa cells, and two-dimensional polyacrylamide gel electrophoresis profiles of [^35^S]-labelled chlamydial proteins from this extract were compared with profiles of chlamydial proteins from the lysate of infected cells. In this way, CT621 was identified. CT621 is a member of a family of proteins containing a domain of unknown function DUF582 that is only found within the genus *Chlamydia*. Immunofluorescence microscopy and immunoblotting demonstrated that CT621 is secreted late in the chlamydial developmental cycle and that it is the first chlamydial protein found to be localized within both the host cell cytoplasm and the nucleus. To determine whether CT621 is secreted through the T3SS, an inhibitor of this apparatus was added to the infection medium, resulting in retention of the protein inside the chlamydiae. Hence, the so far uncharacterized CT621 is a new type III secretion effector protein.

## Introduction

*Chlamydia* are obligate intracellular bacteria with a unique biphasic developmental cycle. The infectious elementary bodies (EB) enter the host cell cytoplasm, where they differentiate into metabolically active reticulate bodies (RB) inside a membrane-bound vacuole, the chlamydial inclusion. The RB multiply and the developmental cycle is completed by asynchronous redifferentiation of RB into EB that are released upon lysis of the host cell (Moulder, 1991; Abdelrahman & Belland, 2005;).

In electron microscopic studies, Matsumoto and colleagues observed projections on the chlamydial surface (Gregory *et al.*, 1979; Matsumoto, 1982;). These projections emerge from beneath depressions of the bacterial plasma membrane, span the periplasmic space and extend out of the outer membrane, where they end with a pointed tip (Nichols *et al.*, 1985). Based on the discovery of genes encoding a type III secretion system (T3SS) in *Chlamydia* (Hsia *et al.*, 1997), the chlamydial surface projections were considered to represent type III secretion (T3S) apparatuses (Bavoil & Hsia, 1998).

The T3SS has certain specific characteristics: firstly, it allows translocation of proteins termed ‘effectors’, across both bacterial membranes and the eukaryotic cell membrane. Secondly, secretion is independent of the general secretory pathway, Sec; however, a noncleavable amino-terminal signal sequence might be localized in the secreted proteins. Finally, the secretion apparatus has a distinctive structure, which indicates that transport through this pathway occurs in a single step (Hueck, 1998; Cornelis, 2006;). When the T3SS is assembled and the effector molecules are synthesized, chaperones are required to actively accomplish the bacterial attack (Feldman & Cornelis, 2003).

The T3SS is essential for numerous gram-negative bacterial species, creating a pathway through which effector proteins can be delivered into eukaryotic cells directly from pathogens such as *Shigella, Salmonella* and *Yersinia* (Hueck, 1998).

While the composition of the T3S apparatus is conserved among bacterial species, a diverse array of effector proteins is delivered to the host cell cytoplasm through this pathway. The effectors modulate or interfere with many of the host cellular functions, both after or during a bacterial invasion (Kubori & Galan, 2003; Zurawski *et al.*, 2006;), and in the same way, they are also used in further development and maintenance of infections (Haraga & Miller, 2003; Okuda *et al.*, 2005;). One functional characteristic common to several of the type III secreted proteins is mimicking the function of host cell proteins, a property that enables pathogenic bacteria to exploit the eukaryotic ubiquitination and small ubiquitin-like modifier systems (Hotson *et al.*, 2003; Angot *et al.*, 2007;).

As for other gram-negative bacteria, the presumed function of the chlamydial T3SS is to establish and maintain an infection. Among the proteins known to be secreted from the chlamydiae inside the inclusions and through the T3SS is the *Chlamydia* outer protein N (CopN), which has a high degree of sequence similarity to *Yersinia* YopN and acts as a regulator of the T3SS. CopN has been demonstrated to be associated with the chlamydial inclusion membrane and can be secreted by the T3SS of *Yersinia* (Fields & Hackstadt, 2000).

Based on the universality of the T3SS among gram-negative bacteria, the ability of chlamydial proteins to be secreted through the T3SS of another pathogen was used to suggest several other new chlamydial T3S effectors. Using this approach, it was proposed that the *Chlamydia* protein associating with death domains (CADD) is a putative substrate for the T3SS (Subtil *et al.*, 2005). A number of additional chlamydial proteins secreted through the T3SS have been described, among them the translocated actin-recruiting phosphoprotein (Tarp) (Clifton *et al.*, 2004) and the family of Inc proteins (Subtil *et al.*, 2001; Rockey *et al.*, 2002;).

It has been demonstrated that the chlamydial T3SS can be blocked by small molecule inhibitors of the *Yersinia* T3SS, belonging to a class of acylated hydrazones of salicylaldehydes. This effect was observed in both *Chlamydia trachomatis* (Muschiol *et al.*, 2006; Wolf *et al.*, 2006;) and *Chlamydia pneumoniae* (Bailey *et al.*, 2007). Treatments with *N*′-(3,5-dibromo-2-hydroxybenzylidene)-4-nitrobenzohydrazide (designated compound 1–C1 or INP0007), INP0400 or INP0010 prevented the secretion of *C. trachomatis* IncA, IncG, Tarp and CADD (Muschiol *et al.*, 2006; Wolf *et al.*, 2006;), and of *C. pneumoniae* IncB and IncC (Bailey *et al.*, 2007). The inhibitors also exhibited a dose-dependent effect on chlamydial growth and development, a finding that might reflect an involvement of T3S effectors in the developmental cycle (Wolf *et al.*, 2006; Peters *et al.*, 2007;).

As described (Hobolt-Pedersen *et al.*, 2006), extraction buffer I from the ProteoExtract™ subcellular proteome extraction kit (Calbiochem, San Diego, CA) can be used to extract the cytoplasm of infected host cells while the chlamydiae are retained. In the analysis of extraction efficiency, the secreted chlamydial protease-like activity factor (CPAF), which has a predicted signal peptidase cleavage site indicative of Sec-dependent secretion (Zhong *et al.*, 2001; Shaw *et al.*, 2002;), was one of the proteins investigated. With antibodies reacting against CPAF, *Chlamydia* major outer membrane protein (MOMP) and *Chlamydia* cytoplasmic protein GroEL, it was demonstrated that whereas the host cytoplasm with its content of CPAF and potentially other secreted chlamydial proteins was extracted with buffer I, the RB and EB membranes remained intact during the procedure.

Comparing the two-dimensional polyacrylamide gel electrophoresis (2D-PAGE) profile of chlamydial proteins from the cytoplasmic extract with that of chlamydial proteins from infected cells, we identified a cleavage product of CT621 being secreted into the cytoplasm of infected host cells. CT621 is a member of the *Chlamydia*-specific family of proteins characterized by having a domain of unknown function, DUF582. With specific antibodies, we visualized the localization of CT621 in both the cytoplasm and the nucleus of infected cells. Based on treatments of infected cells with the T3SS inhibitor *N*′-(3,5-dibromo-2-hydroxybenzylidene)-4-nitrobenzohydrazide, C1, the secretion of CT621 is proposed to occur through the *C. trachomatis* T3S pathway.

## Materials and methods

### Cell culture and infection

HeLa cells [American Type Culture Collection (ATCC), Rockville, MD], found to be free of mycoplasmas by Hoechst no. 33258 staining, were cultivated in Roswell Park Memorial Institute (RPMI)-1640 (Gibo BRL, Grand Island, NY) supplemented with 10% foetal calf serum (heat-inactivated, sterile-filtered) and 10 μg mL^−1^ gentamicin at 37 °C in a 5% CO_2_ atmosphere.

The cells were infected with either 1 or 0.5 inclusion-forming units (IFUs) per cell of *C. trachomatis* serovar L2 (434/Bu) from the ATCC. The medium supplying the infected cells was supplemented with 1 μg mL^−1^ cycloheximide.

### Labelling of chlamydial proteins with [^35^S]-methionine/cysteine

Thirty-six to 38 hours postinfection (hpi), the chlamydial proteins were radioactively labelled in a methionine/cysteine-free RPMI-1640 medium containing 40 μg mL^−1^ cycloheximide and 100 μCi mL^−1^ [^35^S]-methionine/cysteine (Promix from GE Healthcare, Little Chalfont, UK). After the 2-h labelling period, the cells were washed with phosphate-buffered saline before lysis using a 2D-lysis buffer containing 7 M urea, 2 M thiourea, 4% w/v 3-[(3-chloramidopropyl) dimethylammonium]-1-propanesulphonate, 40 mM Tris-base, 65 mM dithioerythritol and 2% v/v Pharmalyte pH 3–10 (GE Healthcare).

### Extraction procedures

Cytoplasmic extraction from HeLa cells infected with *C. trachomatis* L2, 1 IFU per cell, was performed using extraction buffer I from the ProteoExtract™ subcellular proteome extraction kit (Calbiochem) in accordance with the manufacturer's instructions. The infected cells were washed twice and the cytoplasmic fraction was extracted using buffer I supplemented with a protease inhibitor cocktail (provided with the extraction kit) and 1 mM NaVO_3_ for 10 min at 4 °C under gentle agitation. The proteins from the extract were precipitated with 80% acetone for 1 h at −20 °C. Afterwards, the precipitated samples were centrifuged at 15 000 ***g*** (at 0 °C) for 10 min and the pellets were dried.

Extraction of the membrane/organelle and nuclear fractions was performed using extraction buffers II and III from the same extraction kit in accordance with the manufacturer's protocol. Subsequently, the remaining cytoskeletal fraction was dissolved in a sodium dodecyl sulphate (SDS) sample buffer.

### 2D-PAGE

The acetone-precipitated cytoplasmic fraction of infected cells was dissolved in a 2D-lysis buffer, which was also used for the harvest of infected and uninfected cell lysates. The samples were separated by 2D-gel electrophoresis according to Vandahl *et al.* (2001).

Following separation, the gels were fixed in a solution containing 10% acetic acid and 25% isopropanol for 30 min and vacuum dried. The dried gels were placed on phosphorimager screens (GE Healthcare) for 1–2 weeks, exposed on a PhosphorImager 425E (GE Healthcare) and analysed using melanie IV software (Swiss Institute of Bioinformatics, Switzerland).

### MS

2D-gels for mass spectrometric protein identification, loaded with cytoplasmic extract, were washed and vacuum dried without any fixation. Subsequently, the dried gels were exposed to X-ray films (GE Healthcare).

Protein spots of interest were excised from the gels and digested with trypsin as described previously (Vandahl *et al.*, 2001). The digested protein spots were applied for automated nano-liquid chromatography tandem mass spectrometry (LC-MS/MS) analysis using an Ultimate (Dionex, Amsterdam, the Netherlands) in-line connected to an electrospray ionization (ESI) Esquire HCT ion trap (Bruker Daltonics, Bremen, Germany) according to Stucke *et al.* (2007). MS/MS fragmentation spectra were converted to Mascot generic files (mgf) using the automation engine software (version 3.2, Bruker Daltronics) and searched, using a lab-installed version of mascot (version 2.1.0) (http://www.matrixscience.com), against a *C. trachomatis* L2 protein database. mascot's parameter settings were as follows: enzyme, trypsin; variable modifications, carbamidomethyl (C), deamidation (NQ), oxidation (M) and pyro-glu (N-term Q); peptide mass and fragment mass tolerances, ±0.5 Da; maximum number of missed cleavages, 1; and instrument type, ESI-TRAP. Only spectra that exceeded mascot's threshold score for identification (set at a 95% confidence level) were further evaluated.

### Bioinformatics

blastp searches were performed using a BLOSUM62 scoring matrix with gap costs of existence 11 and extension 1 (http://www.ncbi.nlm.nih.gov/BLAST/). The database Pfam 23.0 was used to search for conserved domains. A multiple sequence alignment of CT621 homologues was created in clustalx using the PAM250 amino acid matrix (Higgins & Sharp, 1988; Chenna *et al.*, 2003;). Based on this alignment, a minimum evolutionary phylogenetic tree was constructed using molecular evolutionary genetics analysis (mega) software, version 3.1 (Kumar *et al.*, 2004). Sequences were also compared in a dot plot, with the polydot application from the european molecular biology open software suite (emboss) (Rice *et al.*, 2000).

### Cloning, expression and production of polyclonal antibodies

Using the forward primer GACGACGACAAGATGAACCGTATTCATCGTACACAAGGATC and the reverse primer GAGGAGAAGCCCGGTCTATCTTAAGAGATTACGCGCTAATCC, a PCR of *ct621* was performed on *C. trachomatis* L2 DNA in a GeneAmp PCR system 9600 (Perkin Elmer, Waltham, MA). The PCR programme was composed of 35 cycles of 94 °C for 30 s, 55 °C for 30 s and 72 °C for 1 min, and T4 DNA polymerase (Novagen, Madison, WI) was used. The amplified DNA product was purified using Wizard (Promega, Madison, WI). Following this, cloning and expression of the hexahistidine-tagged protein was performed using the pET-30 LIC Vector Kit (Novagen) according to the manufacturer's instructions. The hexahistidine-tagged fusion protein was purified on Ni^2+^ HiTrap™ chelating HP columns (GE Healthcare) as described (Mygind *et al.*, 1998).

To raise polyclonal antibodies, rabbits were immunized three times intramuscularly with 100 μg fusion protein and incomplete Freund's adjuvant (IFA), and three times intravenously with 100 μg of the fusion protein without IFA (Shaw *et al.*, 2002).

### Immunofluorescence microscopy

HeLa cells, cultivated on coverslips, were infected with 0.5 IFU per cell of *C. trachomatis* L2 and fixed in paraformaldehyde/glutaraldehyde as described (Vandahl *et al.*, 2005). Fixations were performed at different points in time, ranging from 12 to 46 hpi. Primary antibodies, PAb245 against CPAF (Shaw *et al.*, 2002) (1 : 200 dilution) or PAb342 against CT621 (1 : 500 dilution), were used and visualized using fluorescein isothiocyanate (FITC)-conjugated goat anti-rabbit secondary antibodies (1 : 200 dilution) (Jackson ImmunoResearch Laboratories, West Grove, PA). For double-stainings, mouse monoclonal MAb32.2 recognizing MOMP (Birkelund *et al.*, 1988) was used (1 : 40 dilution), together with rhodamine-conjugated goat anti-mouse secondary antibodies (1 : 100 dilution) (Jackson ImmunoResearch Laboratories). Host cell nuclei were visualized using 4′,6-diamidino-2-phenylindole (DAPI).

Images were obtained using a Leitz DMRBE fluorescence microscope (Leica, Heidelburg, Germany) connected to a Sony 3CCD colour video camera. The objective used was a Leica HCX PL APO × 100/1.40 oil lens (Leica).

### Purification of *C. trachomatis* L2 EB

*Chlamydia trachomatis* L2-infected HeLa cell monolayers were scraped off in 4-2-hydroxyethyl-1-piperazineethanesulphonic acid at 38 hpi. After the cell debris was spun down, the supernatant was treated with DNAse and RNAse. *Chlamydia trachomatis* EB were purified by two rounds of density gradient ultracentrifugation through Visipaque (Nycomed, Roskilde, Denmark) gradients, essentially as described in (Schachter & Wyrick, 1994).

### Immunoblotting

Ten percent SDS polyacrylamide gels were loaded with purified *C. trachomatis* EB, lysates of infected and uninfected cells or proteins from each of the four extracted fractions, and electroblotted onto polyvinylidene fluoride membranes. For immuno-detections, PAb342 raised against CT621 was used in a 1 : 500 dilution. Secondary alkaline phosphatase-conjugated goat anti-rabbit antibodies (Sigma-Aldrich, St. Louis, MO) were applied in a dilution of 1 : 30 000.

### Inhibition of the chlamydial T3SS

The T3SS inhibitor *N*′-(3,5-dibromo-2-hydroxybenzylidene)-4-nitrobenzohydrazide, C1 (ChemBridge, San Diego, CA), was dissolved in dimethyl sulphoxide (DMSO), and then 10 or 50 μM of this inhibitor was added at 30, 32 or 34 hpi to the medium of *C. trachomatis* L2-infected HeLa cells (0.5 IFU per cell) cultured on coverslips. The DMSO concentration in the infection medium was maintained below 0.1% in all the experiments. Cells were fixed at 36 hpi and the localization of CT621 and MOMP was visualized using immunofluorescence microscopy with antibodies as described above. Control cells were subjected to a similar treatment, with the exception that pure DMSO was applied, in corresponding concentrations instead of C1.

## Results

### 2D-PAGE analysis of the cytoplasmic extract from infected host cells

**Fig. 1:**
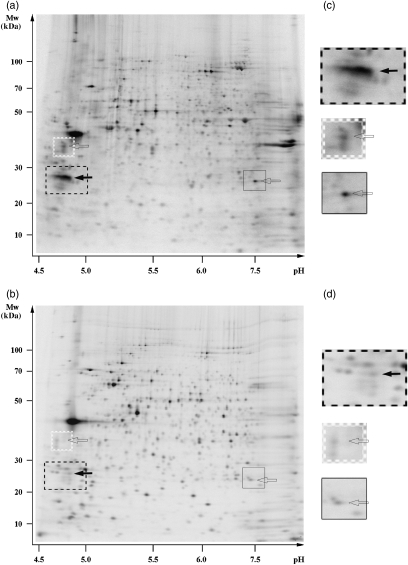
Autoradiographs of 2D-PAGE-separated *Chlamydia trachomatis* L2 proteins labelled from 36 to 38 hpi with [^35^S]-methionine/cysteine in the presence of cycloheximide. (a) Cytoplasmic extract of infected HeLa cells. Hollow arrows indicate CPAF C- and N-terminal parts. The solid arrow indicates the 26-kDa CT621 cleavage product. Regions surrounding these protein spots are boxed. (b) Whole lysate of infected HeLa cells. Boxed regions correspond to the regions mentioned in (a). (c) Enlargement of the boxed regions in (a), picturing the protein spots representing the CT621 cleavage product and both parts of CPAF. (d) Enlargement of the boxed regions in (b).

HeLa cells were infected with *C. trachomatis* L2, and from 36 to 38 hpi, the chlamydial proteins were labelled with [^35^S]-methionine/cysteine in the presence of the eukaryotic protein synthesis inhibitor, cycloheximide. The cells were then either harvested in 2D-lysis buffer or subjected to cytoplasmic extraction with subsequent protein precipitation and resuspension of the pellet in 2D-lysis buffer. The proteins from these samples were separated using 2D-PAGE. Autoradiographs of 2D-PAGE profiles of the separated chlamydial proteins from the cytoplasmic extract (Fig. 1a) and from the lysate of infected cells (Fig. 1b) were compared. Potentially secreted chlamydial proteins were found in higher amounts in the cytoplasmic extract than in the lysate of infected cells because the extraction of cytoplasmic proteins results in their relatively higher concentration. This is indicated by the presence of the secreted N- and C-terminal parts of CPAF with the respective pI 7.3/25 kDa and pI 4.8/36 kDa (Shaw *et al.*, 2002) in both the 2D-PAGE profile of cytoplasmic extract (Fig. 1a and c) and that of the lysate of infected cells (Fig. 1b and d indicated by hollow arrows), with protein spots most visible in the cytoplasmic extract profile (Fig. 1a). The protein content of these spots was verified using MS/MS (data not shown). Further, comparison of the 2D-PAGE profiles revealed another protein spot, with the estimated molecular coordinates pI 4.8/26 kDa, clearly present in the cytoplasmic extract (indicated by a solid arrow inFig. 1a and c), but absent from the lysate of infected host cells (Fig. 1b and d). Because of its abundance in the cytoplasmic extract, this observed protein spot was investigated further.

### Identification of a CT621 fragment

**Fig. 2:**
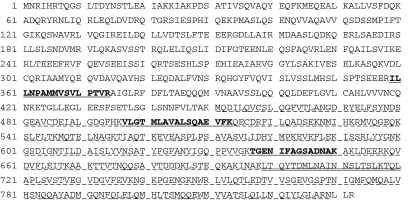
Complete amino acid sequence of the *Chlamydia trachomatis* L2 protein CT621. The DUF582 domain is marked with a dashed line; the leucine zipper is double-underlined, and peptides identified with LC-MS/MS are shown in bold.

The protein spot, which represented a potentially secreted chlamydial protein, was excised and trypsin digested before analysis using automated LC-MS/MS. Peptides from the hypothetical *C. trachomatis* L2 protein, CT621, were identified (bold letters, Fig. 2). In the present paper, we used the annotated *C. trachomatis* D genome for numbering the *C. trachomatis* L2 genes. In the annotated *C. trachomatis* L2 genome, the CT621 gene is CTL0885. A theoretical pI/Mw of pI 4.92/92.7 kDa was calculated for full-length CT621 using the pI/Mw tool on the ExPASy server (http://www.expasy.ch/tools/pi_tool.html). No clear protein spot with these molecular coordinates was found on the autoradiographs (Fig. 1), but a 26-kDa fragment of CT621 was identified (Fig. 2), indicating that the protein present in the host cell cytoplasm was cleaved.

### CT621 homologues

**Fig. 3:**
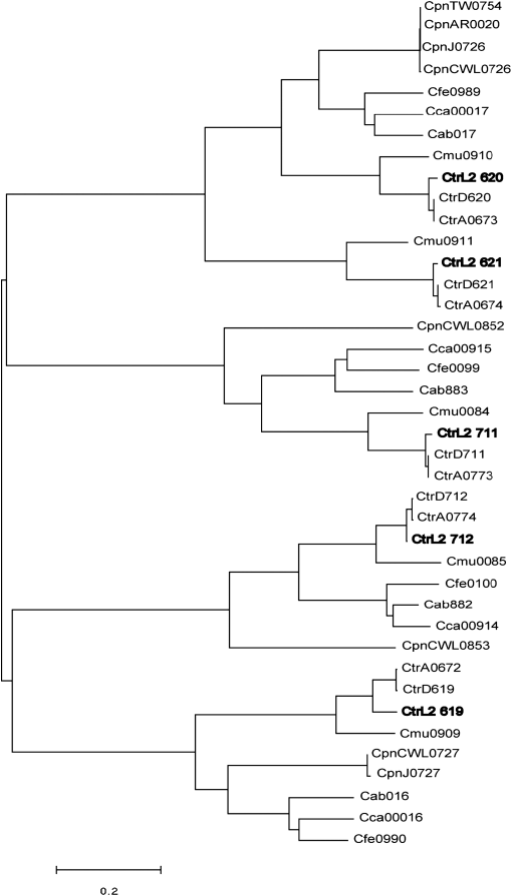
Phylogenetic tree of CT621 protein homologues. Based on a multiple alignment performed in clustalx, a phylogenetic tree was constructed with the minimum evolution method in mega 3.1. The five *Chlamydia trachomatis* serovar L2 homologues are shown in bold. The abbreviations used are listed: *Chlamydia trachomatis* serovars: L2 (CtrL2), D (CtrD) and A (CtrA); *Chlamydia pneumoniae* isolates: CWL029 (CpnCWL), AR39 (CpnAR), J138 (CpnJ), TW-183 (CpnTW), *Chlamydia muridarum* (Cmu), *Chlamydia caviae* (Cca), *Chlamydia felis* (Cfe) and *Chlamydia abortus* (Cab). Where identical sequences are seen, only one isolate is listed.

Four *C. trachomatis* L2 proteins: CT619 (CTL0883), CT620 (CTL0884), CT711 (CTL0080) and CT712 (CTL0081), with molecular weights of 97, 93, 86 and 44 kDa, respectively, showed homology to CT621. Like CT621, these proteins are characterized by having a domain of unknown function, DUF582, in their C-terminal region. In CT621, this domain spans the region from amino acid 451 to 829 (dashed line, Fig. 2). The domain is characterized by being leucine-rich, and a leucine zipper is present in CT620 and CT621 (double-underlined, Fig. 2), indicating that these proteins can form homo- or heterodimers. The remaining three proteins within the DUF582 family do not contain the leucine zipper but are rich in leucine. The CT621 homologous proteins are present in the sequenced genomes of *C. trachomatis* serovars A and D and in other chlamydial species including *Chlamydia abortus, Chlamydia caviae, Chlamydia felis, Chlamydia muridarum* and *C. pneumoniae*, but not in *Parachlamydia* or in other bacteria. Based on a multiple alignment of the related protein sequences performed using clustalx, a minimum evolution phylogenetic tree was constructed using the mega 3.1 software (Fig. 3). From a polydot plot created using emboss, it was confirmed that no rearrangements had occurred. In the phylogenetic tree, it is evident that, based on sequence similarities, the proteins can be divided into four groups: CT620/CT621, CT711, CT712 and CT619. Several chlamydial species are represented in each of these groups, indicating that the genes encoding CT620, CT711, CT712 and CT619 diverged before the different chlamydial species. It is also seen that CT621 diverged from the CT620 group later during evolution than the differentiation into the first four protein groups occurred. The development of CT621 probably occurred as a duplication of the gene encoding CT620 and following differentiation of this into CT621. At that point during evolution, several chlamydial species were already segregated, and therefore CT621 is seen only in *C. trachomatis* and in the closely related *C. muridarum* (Everett *et al.*, 1999).

CT619, CT620 and CT621 are encoded in succession in the chlamydial genome. Nevertheless, their genes are not transcribed as an operon, because CT620 is encoded on the opposite DNA strand (http://stdgen.northwestern.edu).

### Immunofluorescence microscopy

**Fig. 4:**
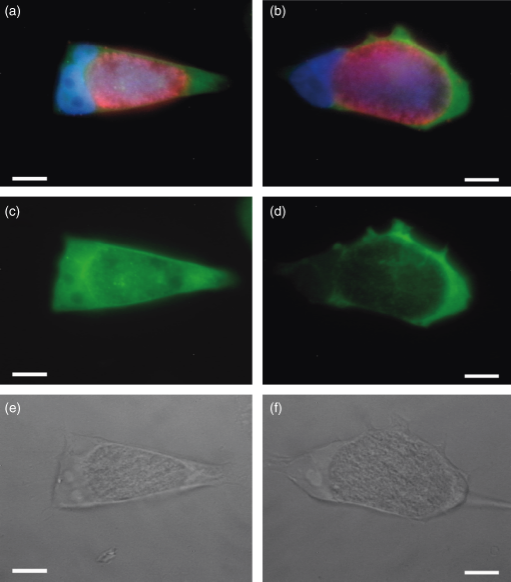
Subcellular localization of CT621 (a, c) and CPAF (b, d) in *Chlamydia trachomatis* L2-infected HeLa cells fixed with paraformaldehyde/glutaraldehyde at 46 hpi. (a, b) CT621 and CPAF are visualized in double stainings using antibodies directed against each of these proteins and against MOMP. The presence of CT621 and CPAF was revealed using FITC (green)-conjugated secondary antibodies and MOMP using rhodamine (red)-conjugated secondary antibodies. DNA was stained with DAPI (blue). (c, d) FITC channel shown separately. (e, f) Corresponding Nomarsky images. Scale bar=10 μm.

In order to compare the subcellular localization of CT621 and CPAF in *C. trachomatis* L2-infected cells fixed at 46 hpi, these proteins were visualized using polyclonal primary antibodies and FITC-conjugated secondary antibodies [Fig. 4a and c (CT621) and Fig. 4b and d (CPAF)]. Localization of the chlamydiae was shown using a MOMP-specific monoclonal antibody and rhodamine-conjugated secondary antibodies, while host nuclei were stained with DAPI (Fig. 4a and b). By displaying the FITC fluorescence alone, the distribution of CT621 and CPAF in the different compartments of the host cell was revealed (Fig. 4c and d). The corresponding Nomarsky images show the localization of the chlamydial inclusions and the host nuclei (Fig. 4e and f). At this point in time, CT621 could be detected in both the cytoplasm and the nuclei of most infected cells, although some CT621 was retained inside the chlamydiae. The retained CT621 is visualized as green spots within the inclusion (Fig. 4c), while the RB surrounding the periphery of the inclusion (Fig. 4a, indicated by red MOMP staining) is devoid of CT621 (Fig. 4c). In contrast, CPAF is seen solely within the host cell cytoplasm, and the inclusion shows no CPAF staining 46 hpi (Fig. 4b and d). These results demonstrate that in contrast to CPAF, which is secreted only into the cytoplasm, CT621 is secreted both into the host cell cytoplasm and the nucleus. However, not all of the chlamydiae had secreted CT621.

During the infection of HeLa cells with *C. trachomatis* L2, cycloheximide was added to the medium to facilitate this process. To ensure that the exposure to cycloheximide was not affecting the HeLa cells to an extent in which degradation of all CT621 in the cytoplasm was prevented, the immunofluorescence analysis was performed again without the eukaryotic protein synthesis inhibitor. CT621 could still be found in both the host cytoplasm and nucleus, indicating that cycloheximide did not have an influence on the levels of secreted CT621 (data not shown).

**Fig. 5:**
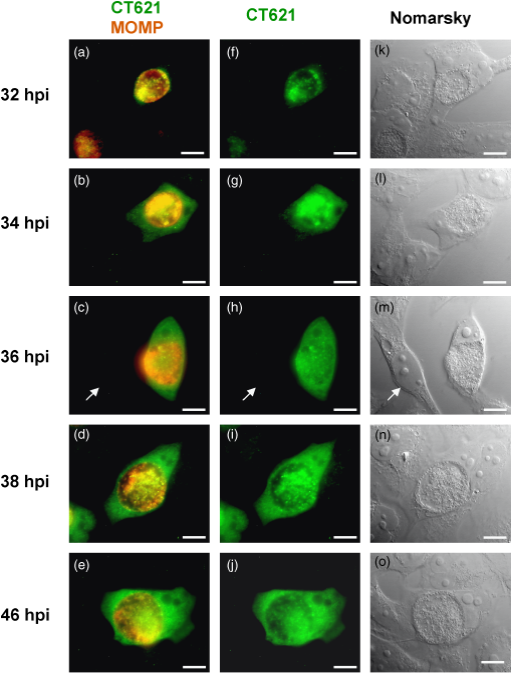
Immunofluorescence microscopy of *Chlamydia trachomatis* L2-infected cells fixed with paraformaldehyde/glutaraldehyde at different points in time after infection. (a–e) Double stainings with primary antibodies against CT621 and MOMP, visualized using FITC (green)-conjugated and rhodamine (red)-conjugated secondary antibodies, respectively. (f–j) FITC channel shown separately. (k–o) Corresponding Nomarsky images. White arrows indicate an uninfected cell in which neither CT621 nor MOMP can be detected (c, h and m). CT621 secretion is initiated between 32 and 34 hpi. Scale bar=10 μm.

To determine at which point during the chlamydial developmental cycle CT621 was secreted, *C. trachomatis* L2-infected cells were fixed at different points in time ranging from 12 to 46 hpi. With immunofluorescence microscopy, it was revealed that no CT621 was secreted before 32 hpi (Fig. 5). Subsequently, at later time points, more and more CT621 was secreted into both host cell cytoplasm and nuclei, and from 38 hpi, CT621 was secreted into almost all infected cells. MOMP was visualized by rhodamine (red) fluorescence and CT621 by FITC (green) fluorescence (Fig. 5a–e). Pictures showing the FITC fluorescence alone (Fig. 5f–j) as well as the corresponding Nomarsky images were also analysed (Fig. 5k–o). At 32 hpi, CT621 could be detected as green spots overlapping with MOMP within the inclusion (Fig. 5a and f). Subsequently at 34 hpi, most of the CT621 was still localized in the chlamydial inclusion, but a small amount of the protein was also observed in the host cytoplasm (Fig. 5b and g). After 36 h of infection, CT621 was secreted and evenly distributed in the host cytoplasm and nucleus as well as in the chlamydial inclusion; however, inside the inclusion, the MOMP staining at the periphery (Fig. 5c) was stronger than the CT621 staining (Fig. 5h). At 38 hpi and until the end of the experiment at 46 hpi, CT621 was still found to be secreted. As expected, neither CT621 nor MOMP was present in the uninfected cells (Fig. 5c, h and m, indicated by arrows). In additional immunofluorescence microscopy analysis, CT621 was also found to be secreted in *C. trachomatis* D-infected HeLa cells.

### Immunoblotting

**Fig. 6:**
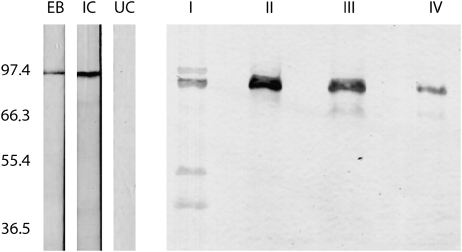
Immunoblotting with CT621-specific antibodies. The inserted scale is created using stained Mark 12 molecular size markers. Polyvinylidene fluoride membranes containing EB: purified *Chlamydia trachomatis* EB; IC, lysate of infected cells; UC, lysate of uninfected cells; I, cytoplasmic extract; II, membrane/organelle/EB fraction; III, nuclear fraction; and IV, cytoskeletal fraction were reacted with anti-CT621 polyclonal antibodies.

Both localization and the size of CT621 were verified by immunoblotting performed on EB, lysates of infected and uninfected cells and on cellular extracts, all harvested at 38 hpi (Fig. 6). Polyclonal CT621-specific antibodies recognized full-length CT621 in EB and in the lysate of infected cells, while no reaction with uninfected HeLa cells was observed. Fractionation of *C. trachomatis* L2-infected cells was performed with extraction buffers I, II and III. Proteins from these fractions and from the remaining cytoskeletal fraction (IV) were separated by SDS-PAGE and subjected to immunoblotting with CT621-specific antibodies (Fig. 6). In the cytoplasmic extract, the full-length protein and fragments of around 40 and 50 kDa were found. Additionally, full-length CT621 bands were observed in all other fractions; however, the strongest staining was seen in the membrane/organelle fraction (II) also containing chlamydial EB, and in the nuclear fraction (III).

### Inhibition of the chlamydial T3SS

**Fig. 7:**
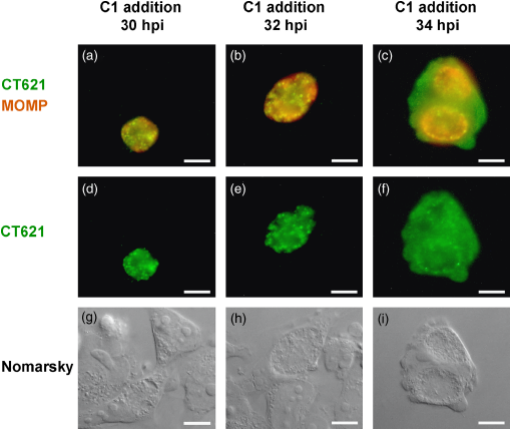
Immunofluorescence microscopy showing the effects of addition of 10 μM of the T3S inhibitor, C1, to *Chlamydia trachomatis* L2-infected cells at 30, 32 or 34 hpi. At 36 hpi, all the cells were fixed with paraformaldehyde/glutaraldehyde. (a–c) Double stainings with primary antibodies against CT621 and MOMP, visualized using FITC (green)-conjugated and rhodamine (red)-conjugated secondary antibodies, respectively. (d–f) FITC channel shown separately. (g–i) Corresponding Nomarsky images. Scale bar=10 μm.

To investigate the CT621 secretion pathway, the T3SS inhibitor, C1, was added to *C. trachomatis* L2-infected cells at different points in time. As described by Wolf *et al.* (2006) and Muschiol *et al.* (2009), C1 affects chlamydial development, and culturing of infected cells in a medium supplemented with C1 for longer time periods results in abnormal, small chlamydial inclusions. However, when added at a later point in time after infection, no such influence of C1 on chlamydial growth was seen (Chellas-Géry *et al.*, 2007). To ensure the least exposure time of chlamydiae to the inhibitor, the addition of C1 was scheduled a few hours before fixation of the infected cells. C1 was added to *C. trachomatis* L2-infected cells at 30, 32 or 34 hpi, before fixation at 36 hpi. The fixation time was set at 36 hpi, because CT621 was clearly visible in the host cell cytoplasm at this time after infection (Fig. 5c and h). C1 concentrations of 10 and 50 μM were used, and after fixation, immunofluorescence microscopy was performed (Fig. 7). When 10 μM C1 was added at 30 or 32 hpi, no CT621 was found in the host cytoplasm at 36 hpi; instead, it was retained within the chlamydiae, where it can be seen as green spots overlapping with MOMP (red) (Fig. 7a, b, d and e). In contrast, if the T3SS inhibitor was added at 34 hpi, CT621 was localized in the host cytoplasm and nucleus (Fig. 7c and f). This indicates that CT621 secretion is initiated between 32 and 34 hpi. Parallel Nomarsky images (Fig. 7g–i) show the inclusions and nuclei within the host cells. There was no indication of impaired integrity of the chlamydial inclusion membrane.

Ten micromolars of C1 was sufficient to cause the inhibition of CT621 secretion, and a similar effect was found with 50 μM C1 at all points in time. Control treatments of infected cells with the C1 solvent, DMSO, did not affect the secretion of CT621.

## Discussion

Based on the results obtained by 2D-PAGE, immunofluorescence microscopy and immunoblotting, a secreted chlamydial protein was identified. Previously, we have shown that extraction buffer I from the ProteoExtract™ subcellular proteome extraction kit can be used to isolate the cytoplasm from *C. trachomatis* L2-infected HeLa cells without rupturing the chlamydial membranes and without extracting the eukaryotic organelles (Hobolt-Pedersen *et al.*, 2006). Abundant chlamydial proteins such as MOMP can be detected when the cytoplasmic fraction is analysed by 2D-PAGE, but even though some rupturing of chlamydiae is unavoidable during the extraction procedure, the chlamydial proteins secreted are still much more concentrated in the cytoplasmic extract than in the lysate of infected cells. This is indicated by the presence of CPAF C- and N-terminal parts, with more intense spots on the 2D-PAGE profile of the cytoplasmic extract compared with the corresponding spots on the profile of the lysate of infected cells (Fig. 1). Comparison of 2D-PAGE profiles also led to the identification of a 26-kDa fragment of CT621. CT621 was first described by Skipp *et al.* (2005), who, using proteomics, identified the protein as being specific for RB. However, none of the other protein family members were described in these investigations.

In the present study, a fragment of CT621 was found in the host cytoplasmic extract, indicating that the protein is cleaved there. This was confirmed using immunoblotting with antibodies raised against CT621 (Fig. 6), together with full-length CT621; different cleavage products were found in the cytoplasmic fraction. In the nuclear extract, however, nearly only full-length CT621 was seen (Fig. 6). Immunofluorescence microscopy showed that CT621 is localized to both the cytoplasm and the nucleus of *C. trachomatis* L2-infected HeLa cells. Thus, CT621 is the first chlamydial protein found in the host nucleus and this observation is strongly supported by the results of the immunoblotting.

As no transformation system exists for the genetic manipulation of chlamydiae, other approaches must be used to study the localization and the potential function of chlamydial proteins. Sisko *et al.* (2006) used the expression of *Chlamydia*-specific genes in yeast to determine whether such proteins could impair various yeast cell functions. They, furthermore, determined whether any of the proteins were translocated to the cell surface, nucleus or other organelles. Of the DUF582 family, four proteins were expressed, but none of them showed nuclear localization. CT620 and CT711 were seen in the yeast cytoplasm, CT712 showed punctuated cytoplasmic staining while CT621 showed cytoplasmic aggregates. That CT621 was not translocated to the yeast nucleus may be explained by the use of an artificial system in which CT621 aggregated within the yeast cytoplasm.

Using immunofluorescence microscopy, it was revealed that the secretion of CT621 is initiated between 32 and 34 hpi. At 34 hpi, a small amount of CT621 was found to be secreted, and this amount increased until 36 hpi, where it was evident that CT621 is secreted into both the host cell cytoplasm and the nuclei of the infected cells. CT621 is secreted late in the chlamydial developmental cycle, indicating that the protein has a function in the later stage of infection. In contrast to what was seen for CPAF, the inclusions contained CT621 at all points in time after secretion could first be observed (Fig. 5). It is therefore possible that secretion of CT621 continues after the initial secretion. Our suggestion that CT621 functions late in the chlamydial developmental cycle finds support in the genomic transcriptional analysis performed by Belland *et al.* (2003). In this study, microarrays were used to investigate gene expression in *C. trachomatis* D at different developmental stages, and it was shown that the expression of the CT621 gene was most profound from 24 hpi and later in the chlamydial developmental cycle. Furthermore, the gene encoding the CT621 homologue, CT712, was defined as a late gene.

Addition of the chlamydial T3SS inhibitor, C1, to *C. trachomatis* L2-infected cells before the initiation of CT621 secretion resulted in retention of the protein inside the chlamydial inclusions. This C1 caused inhibition of CT621 secretion, which indicates that the protein is transported into the eukaryotic host cell through the T3S pathway. That CT621 is secreted through the T3SS is supported by the findings of Subtil *et al.* (2005), who found that in their heterologous *Shigella* system, CT712, which is also a member of the DUF582 family, was secreted by the T3SS. A new hypothesis implies that loss of contact between RB and the inclusion membrane, coupled to T3SS inactivation, triggers the late differentiation of *Chlamydia* (Peters *et al.*, 2007). This could mean that the T3SS is crucial in the regulation of chlamydial growth and development inside the host. The hypothesis is supported by the fact that exposure to inhibitors of the T3SS such as C1 affects the chlamydial development, probably because secretion of certain effectors is altered (Wolf *et al.*, 2006; Bailey *et al.*, 2007; Peters *et al.*, 2007;). In agreement with the described hypothesis, we found indications that CT621 secretion through the T3SS takes place from RB located close to the inclusion membrane (Fig. 4a and c).

CT621 is the first chlamydial protein shown to be translocated to the nucleus of infected cells, even though the nuclear localization, in theory, could be explained by lateral diffusion. However, because CT621 is leucine rich, full-length CT621 is found in the nuclear extract, and because the two secreted fragments of CPAF of 25 and 36 kDa are found only in the cytoplasm of chlamydial-infected cells at the same time as CT621 is found in both inclusions and in host cell cytoplasm and nuclei, we favour that CT621 is translocated to the nucleus. The idea of chlamydial proteins being translocated to the host cell nucleus is supported by the study of Chellas-Géry *et al.* (2007). In this study, it was shown that human Grap2 cyclin D-interacting protein interacts with the chlamydial-specific T3S effector protein CT847, a manipulating interaction that could possibly take place in the host nucleus. Several type III effectors, localized to the host cell nucleus, have been identified in other gram-negative bacteria, and some of these proteins inhibit the expression of host inflammatory response genes. It is possible that CT621 has a similar function and thereby can modulate the host immune defence, although no common amino acid sequence similarities between CT621 and any other such effectors could be identified.

Within the DUF582 domain, CT621 and CT620 have a leucine zipper with four leucine residues at a fixed spacing of seven amino acids (Fig. 2, double-underlined). Leucine zippers form protein dimers, resulting in the formation of helical structures wrapped around each other in a coiled coil, frequently associated with DNA binding (Landschulz *et al.*, 1988; Bornberg-Bauer *et al.*, 1998;). CT621 is not the only type III effector for which a leucine-containing region is characteristic; several other nuclear localizing type III effectors have a leucine-rich region: for example, YopM, IpaH9.8 and SspH1. It is suggested that the leucine-rich regions are related to the nuclear translocation of these proteins. Because CT621 is also localized within the nucleus of infected cells, it can be speculated that the leucine-containing region plays a similar role in this transport.

The DUF582 domain is specific to *Chlamydia*. The CT619-, CT620-, CT711- and CT712-encoding genes are found in all *Chlamydia* species, while the CT621-encoding gene is found only in *C. trachomatis* and *C. muridarum*. The gene family is strictly found in *Chlamydia* and was not detected in the sequenced *Parachlamydia* genome. The encoded proteins must have essential functions because the family is found in all *Chlamydia*. However, the sequence variation between the proteins indicates that they have specific targets. Such targets must be present in all hosts that can be infected by *Chlamydia*. For each protein, major sequence variations are also found between species, indicating that the proteins have adapted to the respective hosts.

## Statement

Reuse of this article is permitted in accordance with the Terms and Conditions set out at http://www3.interscience.wiley.com/authorresources/onlineopen.html
